# 5-Chloro­spiro­[indoline-3,7′-6*H*,7*H*,8*H*-pyrano[3,2-*c*:5,6-*c*′]di[1]benzopyran]-2,6′,8′-trione

**DOI:** 10.1107/S1600536812011932

**Published:** 2012-03-24

**Authors:** Abdulrahman I. Almansour, Raju Suresh Kumar, Natarajan Arumugam, S. Kanagalaksmi, J. Suresh

**Affiliations:** a Department of Chemistry, College of Sciences, King Saud University, PO Box 2455, Riyadh 11451, Saudi Arabia; b Department of Physics, The Madura College, Madurai 625 011, India

## Abstract

The asymmetric unit of the title compound, C_26_H_12_ClNO_6_, consists of two independent mol­ecules. The central pyran rings and both the 1-benzopyran ring systems are nearly planar in both mol­ecules [r.m.s. deviations of pyan rings = 0.0264 (1) and 0.0326 (1) Å for molecules *A* and *B*, respectively; r.m.s. deviations of benzopyran rings = 0.0439 (1) and 0.0105 (1) for molecule *A*, 0.0146 (1) and 0.0262 (1) Å for molecule *B*]. In the crystal, the molecules are linked by C—H⋯O, N—H⋯O and C—H⋯π inter­actions.

## Related literature

The benzopyran structural motif is observed in many biologically active natural products and it plays an important role in binding with various biopolymers, see: Martin & Critchlow (1999 ▶); Teague & Davis (1999 ▶). Spiro indoles are known for their broad spectrum of biological activity, see: Joshi & Jain (1985 ▶). For the pharmacological properties of spiro­[indole-pyran]s, see: Ninamiya (1980 ▶); Kobayashi & Matsuda (1970 ▶).
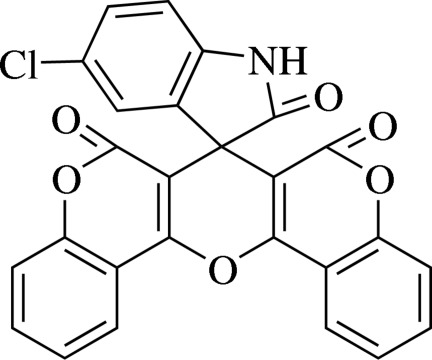


## Experimental

### 

#### Crystal data

C_26_H_12_ClNO_6_
*M* *_r_* = 469.82
Monoclinic, 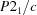

*a* = 16.1910 (3) Å
*b* = 12.9931 (3) Å
*c* = 20.8743 (4) Å
β = 109.091 (1)°
*V* = 4149.83 (15) Å^3^
*Z* = 8
Mo *K*α radiation
μ = 0.23 mm^−1^
*T* = 293 K
0.21 × 0.19 × 0.16 mm

#### Data collection

Bruker Kappa APEXII diffractometer
Absorption correction: multi-scan (*SADABS*; Sheldrick, 1996 ▶) *T*_min_ = 0.973, *T*_max_ = 0.978
35770 measured reflections
11888 independent reflections
8559 reflections with *I* > 2σ(*I*)
*R*_int_ = 0.043

#### Refinement

*R*[*F* ^2^ > 2σ(*F* ^2^)] = 0.079
*wR*(*F* ^2^) = 0.164
*S* = 1.16
11888 reflections
613 parameters
H-atom parameters constrained
Δρ_max_ = 0.64 e Å^−3^
Δρ_min_ = −0.80 e Å^−3^

### 

Data collection: *APEX2* (Bruker, 2004 ▶); cell refinement: *SAINT* (Bruker, 2004 ▶); data reduction: *SAINT*; program(s) used to solve structure: *SHELXS97* (Sheldrick, 2008 ▶); program(s) used to refine structure: *SHELXL97* (Sheldrick, 2008 ▶); molecular graphics: *PLATON* (Spek, 2009 ▶); software used to prepare material for publication: *SHELXL97*.

## Supplementary Material

Crystal structure: contains datablock(s) global, I. DOI: 10.1107/S1600536812011932/ds2182sup1.cif

Structure factors: contains datablock(s) I. DOI: 10.1107/S1600536812011932/ds2182Isup2.hkl

Additional supplementary materials:  crystallographic information; 3D view; checkCIF report

## Figures and Tables

**Table 1:** Hydrogen-bond geometry (Å, °) *Cg*1, *Cg*2 and *Cg*3 are the centroids of the C13*B*–C18*B*, C2*B*–C7*B* and C20*B*–C25*B* rings, respectively.

*D*—H⋯*A*	*D*—H	H⋯*A*	*D*⋯*A*	*D*—H⋯*A*
C22*B*—H22*B*⋯O4*A*	0.93	2.41	3.218 (4)	146
N1*A*—H1*A*⋯O6*B*^i^	0.86	2.08	2.810 (3)	142
N1*B*—H1*B*⋯O5*B*^i^	0.86	2.12	2.968 (3)	169
C5*A*—H5*A*⋯O4*B*^ii^	0.93	2.58	3.227 (4)	127
C5*B*—H5*B*⋯O5*A*^iii^	0.93	2.27	3.196 (4)	173
C14*B*—H14*B*⋯O4*B*^iv^	0.93	2.55	3.035 (4)	113
C17*A*—H17*A*⋯O6*A*^v^	0.93	2.49	3.312 (4)	148
C24*A*—H24*A*⋯Cl2^vi^	0.93	2.69	3.520 (3)	149
C4a—H4a⋯*Cg*1^vi^	0.93	2.95	3.832 (4)	158
C6a—H6a⋯*Cg*2^vi^	0.93	2.74	3.657 (4)	169
C5a—H5a⋯*Cg*3^ii^	0.93	2.88	3.701 (3)	147

Symmetry codes: (i) 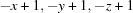; (ii) 
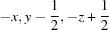
; (iii) 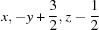; (iv) 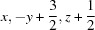; (v) 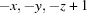; (vi) 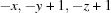.

**Fig. 1.**
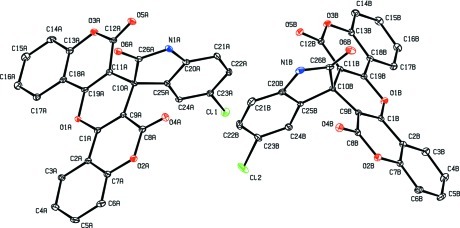
The molecular structure of (I), showing 20% probability displacement ellipsoids and the atom-numbering scheme of molecules A and B.

**Fig. 2.**
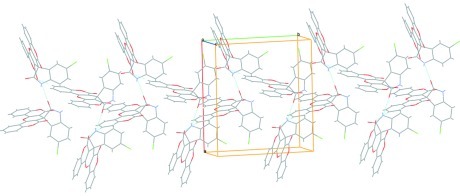
Partial packing diagram of the molecule.

## supplementary crystallographic information

### Comment

Benzopyran is a privileged structural motif observed in many biologically active natural products, and it plays an important role in binding with various biopolymers (Martin & Critchlow (1999)). Spiro indoles are known for their broad spectrum of biological activities (Joshi & Jain (1985)). Of the various spiro indoles, spiro[indole-pyran] system attracted attention due to its interesting pharmacological properties (Ninamiya (1980) and Kobayashi & Matsuda (1970)). The biological importance of these heterocycles in conjunction with our research interest, prompted us to synthesize and report the X-ray studies of the title compound in this paper.

The asymmetric unit of the title compound C_26_ H_12_ CL N O_6_, contains two independent molecules (A and B) with almost identical geometry (Fig.1). The central pyrano ring and both the benzopyran rings are planar. In the Indolin-2-one system, the benzene and pyrrole rings are individually planar in both the molecules and make dihedral angles of 2.25 (1)° in molecule A and 2.32 (1)° in molecule B. The indoline-2-one system is perpendicular to the pyrano ring, as can be seen from the dihedral angle [86.56 (1) in molecule A and 87.07 (1) in molecule B]. The sum of the angles at atom N1 of the indolin-2-one moiety is in accordance with *sp*^2^-hybridization [359.41 (2) and 360.81 (1) in molecules A and B respectively].

The crystal packing shows large number of N—H···O and C—H···O hydrogen bonds. The molecules A and B have intermolecular contacts through N—H···O bonding and generating the crystal structure by large number of zig zag C—H···O hydrogen bonds (Table 1) (Fig.2). In addition there are three weak C—H···π interactions, *viz*., C4a—H4a···*Cg*1^vi^, C6a—H6a···*Cg*2^vi^ and C5a—H5a···*Cg*3 ^ii^, (*Cg*1, *Cg*2 and *Cg*3 are the centroids of the rings C13B—C18B, C2B—C7B and C20B—C25B;symmetry codes are given in Table 1) are observed.

### Experimental

A mixture of 5-chloroindoline-2,3-dione (0.100 g, 0.55 mmol), 4-hydroxy-2*H*-chromen-2-one (0.178 g, 1.10 mmol), and paratoluene sulfonic acid (0.105 g, 0.55 mmol) were dissolved in 5 ml of ethanol:water (1:1 *v*/*v*) and refluxed for 2 h. After completion of the reaction as evident from TLC, the precipitated solid was filtered and washed with water to afford the product which was recrystallized from ethanol to reveal the title compound as colourless crystals. Yield 80%, Melting point 276°C

### Refinement

H atoms were placed at calculated positions and allowed to ride on their carrier atoms with C—H = 0.93 Å, N—H = 0.86 Å and *U*_iso_ = 1.2*U*_eq_(C,*N*) for CH and NH groups.

### Crystal data

**Table:**

C_26_H_12_ClNO_6_	*F*(000) = 1920
*M**_r_* = 469.82	*D*_x_ = 1.504 Mg m^−^^3^
Monoclinic, *P*2_1_/*c*	Mo *K*α radiation, λ = 0.71073 Å
Hall symbol: -P 2ybc	Cell parameters from 2000 reflections
*a* = 16.1910 (3) Å	θ = 2–31°
*b* = 12.9931 (3) Å	µ = 0.23 mm^−^^1^
*c* = 20.8743 (4) Å	*T* = 293 K
β = 109.091 (1)°	Block, colourless
*V* = 4149.83 (15) Å^3^	0.21 × 0.19 × 0.16 mm
*Z* = 8	

### Data collection

**Table:**

Bruker Kappa APEXII diffractometer	11888 independent reflections
Radiation source: fine-focus sealed tube	8559 reflections with *I* > 2σ(*I*)
Graphite monochromator	*R*_int_ = 0.043
Detector resolution: 0 pixels mm^-1^	θ_max_ = 29.9°, θ_min_ = 1.9°
ω and φ scans	*h* = −22→22
Absorption correction: multi-scan (*SADABS*; Sheldrick, 1996)	*k* = −18→12
*T*_min_ = 0.973, *T*_max_ = 0.978	*l* = −29→21
35770 measured reflections	

### Refinement

**Table:**

Refinement on *F*^2^	Primary atom site location: structure-invariant direct methods
Least-squares matrix: full	Secondary atom site location: difference Fourier map
*R*[*F*^2^ > 2σ(*F*^2^)] = 0.079	Hydrogen site location: inferred from neighbouring sites
*wR*(*F*^2^) = 0.164	H-atom parameters constrained
*S* = 1.16	*w* = 1/[σ^2^(*F*_o_^2^) + (0.0208*P*)^2^ + 11.0869*P*] where *P* = (*F*_o_^2^ + 2*F*_c_^2^)/3
11888 reflections	(Δ/σ)_max_ = 0.001
613 parameters	Δρ_max_ = 0.64 e Å^−^^3^
0 restraints	Δρ_min_ = −0.80 e Å^−^^3^

### Special details

**Table:**

Geometry. All e.s.d.'s (except the e.s.d. in the dihedral angle between two l.s. planes) are estimated using the full covariance matrix. The cell e.s.d.'s are taken into account individually in the estimation of e.s.d.'s in distances, angles and torsion angles; correlations between e.s.d.'s in cell parameters are only used when they are defined by crystal symmetry. An approximate (isotropic) treatment of cell e.s.d.'s is used for estimating e.s.d.'s involving l.s. planes.
Refinement. Refinement of *F*^2^ against ALL reflections. The weighted *R*-factor *wR* and goodness of fit *S* are based on *F*^2^, conventional *R*-factors *R* are based on *F*, with *F* set to zero for negative *F*^2^. The threshold expression of *F*^2^ > σ(*F*^2^) is used only for calculating *R*-factors(gt) *etc*. and is not relevant to the choice of reflections for refinement. *R*-factors based on *F*^2^ are statistically about twice as large as those based on *F*, and *R*- factors based on ALL data will be even larger.

### Fractional atomic coordinates and isotropic or equivalent isotropic displacement parameters (Å^2^)

**Table:**

	*x*	*y*	*z*	*U*_iso_*/*U*_eq_	
C1A	−0.01322 (18)	0.1554 (2)	0.45426 (14)	0.0166 (6)	
C1B	0.38576 (17)	0.9265 (2)	0.43954 (14)	0.0164 (6)	
C2A	−0.07950 (18)	0.1436 (2)	0.38862 (14)	0.0166 (6)	
C2B	0.36099 (18)	1.0100 (2)	0.39167 (15)	0.0183 (6)	
C3A	−0.16246 (19)	0.1007 (2)	0.37875 (15)	0.0201 (6)	
H3A	−0.1771	0.0759	0.4154	0.024*	
C3B	0.3500 (2)	1.1115 (3)	0.40901 (17)	0.0246 (7)	
H3B	0.3620	1.1296	0.4542	0.029*	
C4A	−0.2219 (2)	0.0956 (3)	0.31394 (16)	0.0249 (7)	
H4A	−0.2770	0.0677	0.3071	0.030*	
C4B	0.3216 (2)	1.1844 (3)	0.35926 (18)	0.0309 (8)	
H4B	0.3142	1.2520	0.3710	0.037*	
C5A	−0.2002 (2)	0.1320 (3)	0.25879 (16)	0.0256 (7)	
H5A	−0.2410	0.1282	0.2155	0.031*	
C5B	0.3038 (2)	1.1588 (3)	0.29149 (18)	0.0310 (8)	
H5B	0.2845	1.2092	0.2583	0.037*	
C6A	−0.1183 (2)	0.1737 (3)	0.26765 (16)	0.0246 (7)	
H6A	−0.1036	0.1975	0.2308	0.030*	
C6B	0.3145 (2)	1.0584 (3)	0.27312 (17)	0.0276 (7)	
H6B	0.3027	1.0407	0.2278	0.033*	
C7A	−0.05910 (19)	0.1790 (2)	0.33282 (15)	0.0191 (6)	
C7B	0.34329 (18)	0.9847 (2)	0.32374 (15)	0.0197 (6)	
C8A	0.08564 (19)	0.2313 (2)	0.40185 (15)	0.0193 (6)	
C8B	0.37897 (18)	0.8072 (2)	0.34880 (15)	0.0186 (6)	
C9A	0.06541 (18)	0.1972 (2)	0.46159 (14)	0.0157 (6)	
C9B	0.39228 (18)	0.8291 (2)	0.42029 (14)	0.0160 (6)	
C10A	0.13538 (17)	0.2133 (2)	0.53026 (14)	0.0161 (6)	
C10B	0.40791 (17)	0.7387 (2)	0.46806 (14)	0.0138 (5)	
C11A	0.10273 (18)	0.1650 (2)	0.58321 (14)	0.0169 (6)	
C11B	0.42934 (17)	0.7793 (2)	0.53972 (14)	0.0152 (6)	
C12A	0.16297 (19)	0.1617 (2)	0.65275 (15)	0.0192 (6)	
C12B	0.45802 (18)	0.7069 (2)	0.59557 (15)	0.0175 (6)	
C13A	0.0461 (2)	0.0984 (2)	0.68946 (15)	0.0204 (6)	
C13B	0.46670 (19)	0.8459 (2)	0.67352 (15)	0.0185 (6)	
C14A	0.0207 (2)	0.0695 (3)	0.74419 (16)	0.0263 (7)	
H14A	0.0584	0.0776	0.7883	0.032*	
C14B	0.4861 (2)	0.8739 (3)	0.74104 (16)	0.0241 (7)	
H14B	0.5053	0.8253	0.7754	0.029*	
C15A	−0.0614 (2)	0.0285 (3)	0.73204 (17)	0.0294 (8)	
H15A	−0.0792	0.0090	0.7684	0.035*	
C15B	0.4764 (2)	0.9760 (3)	0.75593 (16)	0.0268 (7)	
H15B	0.4896	0.9962	0.8009	0.032*	
C16A	−0.1185 (2)	0.0157 (3)	0.66579 (17)	0.0261 (7)	
H16A	−0.1731	−0.0138	0.6583	0.031*	
C16B	0.4471 (2)	1.0488 (3)	0.70474 (16)	0.0251 (7)	
H16B	0.4398	1.1168	0.7157	0.030*	
C17A	−0.0938 (2)	0.0470 (2)	0.61155 (16)	0.0206 (6)	
H17A	−0.1321	0.0397	0.5675	0.025*	
C17B	0.4287 (2)	1.0207 (2)	0.63795 (16)	0.0215 (6)	
H17B	0.4096	1.0696	0.6038	0.026*	
C18A	−0.01047 (19)	0.0899 (2)	0.62302 (15)	0.0176 (6)	
C18B	0.43903 (18)	0.9177 (2)	0.62167 (14)	0.0169 (6)	
C19A	0.02145 (18)	0.1273 (2)	0.57045 (14)	0.0162 (6)	
C19B	0.42236 (17)	0.8796 (2)	0.55366 (14)	0.0160 (6)	
C20A	0.25197 (18)	0.3343 (2)	0.55651 (14)	0.0177 (6)	
C20B	0.3603 (2)	0.5689 (2)	0.43396 (15)	0.0228 (7)	
C21A	0.29529 (19)	0.4267 (2)	0.57562 (15)	0.0198 (6)	
H21A	0.3547	0.4323	0.5818	0.024*	
C21B	0.3040 (3)	0.4868 (3)	0.41425 (17)	0.0345 (9)	
H21B	0.3241	0.4223	0.4068	0.041*	
C22A	0.2481 (2)	0.5107 (2)	0.58531 (15)	0.0209 (6)	
H22A	0.2757	0.5737	0.5983	0.025*	
C22B	0.2164 (3)	0.5033 (3)	0.40578 (17)	0.0385 (10)	
H22B	0.1769	0.4491	0.3928	0.046*	
C23A	0.15950 (19)	0.5007 (2)	0.57560 (15)	0.0192 (6)	
C23B	0.1875 (2)	0.5995 (3)	0.41641 (16)	0.0293 (8)	
C24A	0.11517 (18)	0.4083 (2)	0.55557 (14)	0.0165 (6)	
H24A	0.0555	0.4032	0.5484	0.020*	
C24B	0.24430 (19)	0.6822 (3)	0.43870 (15)	0.0217 (6)	
H24B	0.2245	0.7463	0.4472	0.026*	
C25A	0.16235 (18)	0.3248 (2)	0.54681 (14)	0.0161 (6)	
C25B	0.33124 (18)	0.6645 (2)	0.44753 (14)	0.0161 (6)	
C26A	0.22395 (18)	0.1639 (2)	0.53016 (15)	0.0187 (6)	
C26B	0.48296 (19)	0.6683 (3)	0.46144 (14)	0.0195 (6)	
N1A	0.28499 (15)	0.2396 (2)	0.54516 (13)	0.0204 (5)	
H1A	0.3384	0.2301	0.5474	0.024*	
N1B	0.44963 (18)	0.5739 (2)	0.44268 (13)	0.0250 (6)	
H1B	0.4798	0.5225	0.4367	0.030*	
O1A	−0.03879 (12)	0.12121 (16)	0.50672 (10)	0.0179 (4)	
O1B	0.39990 (13)	0.95531 (16)	0.50537 (10)	0.0193 (4)	
O2A	0.02128 (13)	0.22147 (17)	0.33975 (10)	0.0213 (5)	
O2B	0.35248 (14)	0.88599 (17)	0.30331 (10)	0.0219 (5)	
O3A	0.12997 (14)	0.13474 (18)	0.70343 (10)	0.0232 (5)	
O3B	0.47610 (14)	0.74300 (17)	0.66028 (10)	0.0215 (5)	
O4A	0.15420 (14)	0.26765 (18)	0.40284 (11)	0.0245 (5)	
O4B	0.38904 (15)	0.72334 (18)	0.32780 (11)	0.0250 (5)	
O5A	0.23954 (14)	0.18162 (19)	0.66861 (11)	0.0263 (5)	
O5B	0.46879 (14)	0.61531 (17)	0.58930 (11)	0.0218 (5)	
O6A	0.23407 (14)	0.07422 (17)	0.51840 (11)	0.0231 (5)	
O6B	0.55785 (14)	0.6966 (2)	0.47220 (12)	0.0310 (6)	
Cl1	0.10234 (5)	0.60744 (6)	0.58955 (5)	0.03013 (19)	
Cl2	0.07605 (6)	0.61968 (10)	0.40081 (5)	0.0485 (3)	

### Atomic displacement parameters (Å^2^)

**Table:**

	*U*^11^	*U*^22^	*U*^33^	*U*^12^	*U*^13^	*U*^23^
C1A	0.0203 (13)	0.0121 (14)	0.0164 (13)	0.0034 (11)	0.0048 (11)	−0.0011 (11)
C1B	0.0121 (12)	0.0210 (15)	0.0155 (13)	−0.0015 (11)	0.0034 (10)	−0.0008 (12)
C2A	0.0184 (13)	0.0122 (14)	0.0165 (13)	0.0032 (11)	0.0023 (11)	−0.0028 (11)
C2B	0.0159 (13)	0.0202 (15)	0.0179 (14)	0.0013 (11)	0.0044 (11)	0.0003 (12)
C3A	0.0199 (14)	0.0178 (15)	0.0193 (14)	0.0013 (12)	0.0020 (11)	−0.0020 (12)
C3B	0.0297 (16)	0.0223 (16)	0.0230 (15)	0.0034 (13)	0.0104 (13)	−0.0003 (13)
C4A	0.0187 (14)	0.0256 (18)	0.0252 (16)	0.0015 (13)	0.0001 (12)	−0.0026 (13)
C4B	0.0375 (19)	0.0232 (18)	0.0343 (19)	0.0066 (15)	0.0150 (16)	0.0058 (15)
C5A	0.0211 (15)	0.0285 (18)	0.0189 (15)	0.0057 (13)	−0.0049 (12)	−0.0040 (13)
C5B	0.0341 (18)	0.0288 (19)	0.0290 (18)	0.0092 (15)	0.0087 (15)	0.0146 (15)
C6A	0.0287 (16)	0.0255 (17)	0.0176 (14)	0.0083 (14)	0.0047 (13)	0.0006 (13)
C6B	0.0271 (16)	0.034 (2)	0.0208 (16)	0.0021 (14)	0.0071 (13)	0.0067 (14)
C7A	0.0195 (14)	0.0140 (14)	0.0213 (15)	0.0036 (11)	0.0032 (12)	−0.0024 (12)
C7B	0.0159 (13)	0.0205 (16)	0.0220 (15)	−0.0010 (12)	0.0052 (11)	0.0001 (12)
C8A	0.0226 (15)	0.0149 (15)	0.0186 (14)	0.0025 (12)	0.0043 (12)	−0.0023 (12)
C8B	0.0163 (13)	0.0211 (16)	0.0196 (14)	−0.0039 (11)	0.0075 (11)	−0.0020 (12)
C9A	0.0159 (13)	0.0136 (14)	0.0142 (13)	0.0017 (11)	0.0002 (10)	−0.0002 (11)
C9B	0.0159 (13)	0.0163 (14)	0.0157 (13)	−0.0009 (11)	0.0050 (11)	−0.0010 (11)
C10A	0.0130 (12)	0.0148 (14)	0.0171 (13)	0.0005 (11)	0.0005 (11)	−0.0034 (11)
C10B	0.0129 (12)	0.0140 (14)	0.0133 (13)	−0.0002 (10)	0.0025 (10)	−0.0028 (11)
C11A	0.0184 (13)	0.0136 (14)	0.0165 (14)	0.0019 (11)	0.0028 (11)	−0.0032 (11)
C11B	0.0122 (12)	0.0172 (14)	0.0155 (13)	−0.0003 (11)	0.0036 (10)	−0.0014 (11)
C12A	0.0209 (14)	0.0158 (15)	0.0152 (13)	0.0020 (12)	−0.0020 (11)	−0.0018 (11)
C12B	0.0163 (13)	0.0184 (15)	0.0181 (14)	0.0005 (11)	0.0059 (11)	0.0008 (12)
C13A	0.0239 (15)	0.0161 (15)	0.0193 (14)	0.0039 (12)	0.0045 (12)	−0.0022 (12)
C13B	0.0193 (13)	0.0189 (15)	0.0176 (14)	−0.0021 (12)	0.0063 (11)	−0.0035 (12)
C14A	0.0308 (17)	0.0297 (19)	0.0150 (14)	0.0064 (14)	0.0029 (13)	0.0009 (13)
C14B	0.0256 (16)	0.0293 (18)	0.0170 (14)	−0.0015 (13)	0.0062 (12)	0.0001 (13)
C15A	0.0354 (18)	0.032 (2)	0.0237 (16)	0.0046 (15)	0.0142 (14)	0.0041 (15)
C15B	0.0273 (16)	0.036 (2)	0.0180 (15)	−0.0039 (14)	0.0094 (13)	−0.0089 (14)
C16A	0.0231 (15)	0.0278 (18)	0.0294 (17)	0.0020 (13)	0.0112 (13)	0.0024 (14)
C16B	0.0255 (16)	0.0260 (18)	0.0247 (16)	−0.0013 (13)	0.0094 (13)	−0.0093 (14)
C17A	0.0215 (14)	0.0187 (15)	0.0208 (15)	0.0040 (12)	0.0061 (12)	−0.0003 (12)
C17B	0.0230 (15)	0.0195 (16)	0.0214 (15)	0.0020 (12)	0.0064 (12)	−0.0016 (12)
C18A	0.0212 (14)	0.0138 (14)	0.0162 (13)	0.0039 (11)	0.0040 (11)	−0.0015 (11)
C18B	0.0147 (12)	0.0199 (15)	0.0151 (13)	0.0001 (11)	0.0037 (10)	−0.0016 (11)
C19A	0.0190 (13)	0.0128 (14)	0.0131 (13)	0.0040 (11)	0.0001 (11)	−0.0009 (11)
C19B	0.0138 (12)	0.0183 (15)	0.0150 (13)	0.0017 (11)	0.0036 (10)	0.0012 (11)
C20A	0.0172 (13)	0.0214 (16)	0.0128 (13)	0.0021 (12)	0.0028 (11)	−0.0001 (11)
C20B	0.0309 (16)	0.0190 (16)	0.0129 (13)	0.0017 (13)	−0.0003 (12)	0.0008 (12)
C21A	0.0156 (13)	0.0221 (16)	0.0197 (14)	−0.0039 (12)	0.0030 (11)	−0.0022 (12)
C21B	0.055 (2)	0.0192 (17)	0.0197 (16)	−0.0039 (16)	−0.0009 (16)	0.0003 (14)
C22A	0.0256 (15)	0.0165 (15)	0.0183 (14)	−0.0061 (12)	0.0039 (12)	−0.0020 (12)
C22B	0.049 (2)	0.037 (2)	0.0187 (16)	−0.0265 (18)	−0.0026 (15)	0.0036 (15)
C23A	0.0241 (15)	0.0153 (15)	0.0163 (14)	0.0055 (12)	0.0040 (12)	−0.0011 (11)
C23B	0.0210 (15)	0.047 (2)	0.0162 (15)	−0.0158 (15)	0.0008 (12)	0.0042 (15)
C24A	0.0148 (12)	0.0186 (15)	0.0143 (13)	0.0019 (11)	0.0022 (10)	−0.0005 (11)
C24B	0.0216 (14)	0.0281 (17)	0.0148 (14)	−0.0030 (13)	0.0052 (11)	0.0020 (12)
C25A	0.0144 (12)	0.0161 (14)	0.0153 (13)	−0.0015 (11)	0.0015 (10)	−0.0015 (11)
C25B	0.0167 (13)	0.0173 (15)	0.0103 (12)	−0.0017 (11)	−0.0010 (10)	0.0010 (11)
C26A	0.0173 (13)	0.0201 (16)	0.0154 (13)	0.0037 (12)	0.0009 (11)	−0.0011 (12)
C26B	0.0193 (14)	0.0253 (16)	0.0119 (13)	0.0088 (12)	0.0023 (11)	0.0003 (12)
N1A	0.0124 (11)	0.0210 (14)	0.0261 (13)	0.0031 (10)	0.0039 (10)	−0.0051 (11)
N1B	0.0331 (15)	0.0192 (14)	0.0196 (13)	0.0118 (11)	0.0044 (11)	−0.0027 (11)
O1A	0.0158 (9)	0.0202 (11)	0.0150 (10)	−0.0020 (8)	0.0013 (8)	0.0001 (8)
O1B	0.0254 (11)	0.0154 (10)	0.0165 (10)	0.0042 (9)	0.0062 (8)	−0.0013 (8)
O2A	0.0227 (11)	0.0213 (12)	0.0177 (10)	0.0002 (9)	0.0039 (8)	0.0007 (9)
O2B	0.0281 (11)	0.0219 (12)	0.0154 (10)	−0.0015 (9)	0.0067 (9)	−0.0010 (9)
O3A	0.0233 (11)	0.0259 (12)	0.0163 (10)	−0.0015 (9)	0.0007 (8)	−0.0023 (9)
O3B	0.0282 (11)	0.0198 (11)	0.0154 (10)	0.0018 (9)	0.0056 (9)	0.0004 (9)
O4A	0.0230 (11)	0.0251 (12)	0.0259 (12)	−0.0039 (9)	0.0087 (9)	−0.0026 (10)
O4B	0.0314 (12)	0.0235 (12)	0.0214 (11)	−0.0007 (10)	0.0105 (9)	−0.0055 (9)
O5A	0.0213 (11)	0.0292 (13)	0.0226 (11)	−0.0016 (9)	−0.0009 (9)	−0.0052 (10)
O5B	0.0286 (11)	0.0167 (11)	0.0202 (10)	0.0037 (9)	0.0080 (9)	0.0030 (9)
O6A	0.0218 (10)	0.0187 (11)	0.0252 (11)	0.0047 (9)	0.0027 (9)	−0.0050 (9)
O6B	0.0177 (11)	0.0433 (15)	0.0327 (13)	0.0078 (10)	0.0094 (10)	−0.0036 (12)
Cl1	0.0336 (4)	0.0175 (4)	0.0383 (5)	0.0069 (3)	0.0103 (4)	−0.0062 (3)
Cl2	0.0240 (4)	0.0915 (9)	0.0294 (5)	−0.0267 (5)	0.0078 (3)	−0.0005 (5)

### Geometric parameters (Å, º)

**Table:**

C1A—C9A	1.346 (4)	C13A—C18A	1.394 (4)
C1A—O1A	1.366 (3)	C13B—O3B	1.384 (4)
C1A—C2A	1.446 (4)	C13B—C18B	1.387 (4)
C1B—C9B	1.342 (4)	C13B—C14B	1.388 (4)
C1B—O1B	1.369 (3)	C14A—C15A	1.376 (5)
C1B—C2B	1.440 (4)	C14A—H14A	0.9300
C2A—C7A	1.389 (4)	C14B—C15B	1.382 (5)
C2A—C3A	1.406 (4)	C14B—H14B	0.9300
C2B—C7B	1.391 (4)	C15A—C16A	1.399 (5)
C2B—C3B	1.394 (4)	C15A—H15A	0.9300
C3A—C4A	1.382 (4)	C15B—C16B	1.389 (5)
C3A—H3A	0.9300	C15B—H15B	0.9300
C3B—C4B	1.369 (5)	C16A—C17A	1.379 (4)
C3B—H3B	0.9300	C16A—H16A	0.9300
C4A—C5A	1.392 (5)	C16B—C17B	1.377 (4)
C4A—H4A	0.9300	C16B—H16B	0.9300
C4B—C5B	1.388 (5)	C17A—C18A	1.406 (4)
C4B—H4B	0.9300	C17A—H17A	0.9300
C5A—C6A	1.387 (5)	C17B—C18B	1.404 (4)
C5A—H5A	0.9300	C17B—H17B	0.9300
C5B—C6B	1.387 (5)	C18A—C19A	1.442 (4)
C5B—H5B	0.9300	C18B—C19B	1.443 (4)
C6A—C7A	1.386 (4)	C19A—O1A	1.371 (3)
C6A—H6A	0.9300	C19B—O1B	1.369 (3)
C6B—C7B	1.389 (4)	C20A—C21A	1.382 (4)
C6B—H6B	0.9300	C20A—N1A	1.392 (4)
C7A—O2A	1.377 (4)	C20A—C25A	1.403 (4)
C7B—O2B	1.374 (4)	C20B—C21B	1.376 (5)
C8A—O4A	1.200 (4)	C20B—C25B	1.390 (4)
C8A—O2A	1.378 (3)	C20B—N1B	1.399 (4)
C8A—C9A	1.458 (4)	C21A—C22A	1.385 (4)
C8B—O4B	1.206 (4)	C21A—H21A	0.9300
C8B—O2B	1.366 (4)	C21B—C22B	1.388 (6)
C8B—C9B	1.464 (4)	C21B—H21B	0.9300
C9A—C10A	1.523 (4)	C22A—C23A	1.388 (4)
C9B—C10B	1.508 (4)	C22A—H22A	0.9300
C10A—C11A	1.509 (4)	C22B—C23B	1.377 (6)
C10A—C25A	1.520 (4)	C22B—H22B	0.9300
C10A—C26A	1.572 (4)	C23A—C24A	1.391 (4)
C10B—C11B	1.516 (4)	C23A—Cl1	1.744 (3)
C10B—C25B	1.518 (4)	C23B—C24B	1.392 (5)
C10B—C26B	1.563 (4)	C23B—Cl2	1.746 (3)
C11A—C19A	1.347 (4)	C24A—C25A	1.372 (4)
C11A—C12A	1.461 (4)	C24A—H24A	0.9300
C11B—C19B	1.348 (4)	C24B—C25B	1.378 (4)
C11B—C12B	1.451 (4)	C24B—H24B	0.9300
C12A—O5A	1.202 (4)	C26A—O6A	1.214 (4)
C12A—O3A	1.377 (4)	C26A—N1A	1.356 (4)
C12B—O5B	1.216 (4)	C26B—O6B	1.216 (4)
C12B—O3B	1.368 (4)	C26B—N1B	1.346 (4)
C13A—O3A	1.376 (4)	N1A—H1A	0.8600
C13A—C14A	1.387 (4)	N1B—H1B	0.8600
			
C9A—C1A—O1A	124.3 (3)	C15B—C14B—C13B	118.3 (3)
C9A—C1A—C2A	122.1 (3)	C15B—C14B—H14B	120.8
O1A—C1A—C2A	113.6 (2)	C13B—C14B—H14B	120.8
C9B—C1B—O1B	123.7 (3)	C14A—C15A—C16A	120.9 (3)
C9B—C1B—C2B	122.3 (3)	C14A—C15A—H15A	119.6
O1B—C1B—C2B	114.0 (3)	C16A—C15A—H15A	119.6
C7A—C2A—C3A	119.1 (3)	C14B—C15B—C16B	121.0 (3)
C7A—C2A—C1A	117.0 (3)	C14B—C15B—H15B	119.5
C3A—C2A—C1A	123.9 (3)	C16B—C15B—H15B	119.5
C7B—C2B—C3B	119.1 (3)	C17A—C16A—C15A	120.1 (3)
C7B—C2B—C1B	116.5 (3)	C17A—C16A—H16A	119.9
C3B—C2B—C1B	124.2 (3)	C15A—C16A—H16A	119.9
C4A—C3A—C2A	119.2 (3)	C17B—C16B—C15B	120.3 (3)
C4A—C3A—H3A	120.4	C17B—C16B—H16B	119.9
C2A—C3A—H3A	120.4	C15B—C16B—H16B	119.9
C4B—C3B—C2B	119.8 (3)	C16A—C17A—C18A	119.8 (3)
C4B—C3B—H3B	120.1	C16A—C17A—H17A	120.1
C2B—C3B—H3B	120.1	C18A—C17A—H17A	120.1
C3A—C4A—C5A	120.7 (3)	C16B—C17B—C18B	119.6 (3)
C3A—C4A—H4A	119.7	C16B—C17B—H17B	120.2
C5A—C4A—H4A	119.7	C18B—C17B—H17B	120.2
C3B—C4B—C5B	120.9 (3)	C13A—C18A—C17A	118.8 (3)
C3B—C4B—H4B	119.5	C13A—C18A—C19A	116.6 (3)
C5B—C4B—H4B	119.5	C17A—C18A—C19A	124.5 (3)
C6A—C5A—C4A	120.8 (3)	C13B—C18B—C17B	119.0 (3)
C6A—C5A—H5A	119.6	C13B—C18B—C19B	116.5 (3)
C4A—C5A—H5A	119.6	C17B—C18B—C19B	124.5 (3)
C6B—C5B—C4B	120.2 (3)	C11A—C19A—O1A	123.5 (3)
C6B—C5B—H5B	119.9	C11A—C19A—C18A	122.9 (3)
C4B—C5B—H5B	119.9	O1A—C19A—C18A	113.6 (2)
C7A—C6A—C5A	118.3 (3)	C11B—C19B—O1B	123.8 (3)
C7A—C6A—H6A	120.8	C11B—C19B—C18B	122.8 (3)
C5A—C6A—H6A	120.8	O1B—C19B—C18B	113.4 (3)
C5B—C6B—C7B	118.7 (3)	C21A—C20A—N1A	129.0 (3)
C5B—C6B—H6B	120.7	C21A—C20A—C25A	121.4 (3)
C7B—C6B—H6B	120.7	N1A—C20A—C25A	109.6 (3)
O2A—C7A—C6A	116.7 (3)	C21B—C20B—C25B	121.3 (3)
O2A—C7A—C2A	121.4 (3)	C21B—C20B—N1B	129.0 (3)
C6A—C7A—C2A	121.9 (3)	C25B—C20B—N1B	109.7 (3)
O2B—C7B—C6B	116.8 (3)	C20A—C21A—C22A	118.4 (3)
O2B—C7B—C2B	122.0 (3)	C20A—C21A—H21A	120.8
C6B—C7B—C2B	121.2 (3)	C22A—C21A—H21A	120.8
O4A—C8A—O2A	117.6 (3)	C20B—C21B—C22B	117.8 (4)
O4A—C8A—C9A	124.8 (3)	C20B—C21B—H21B	121.1
O2A—C8A—C9A	117.6 (3)	C22B—C21B—H21B	121.1
O4B—C8B—O2B	118.3 (3)	C21A—C22A—C23A	119.9 (3)
O4B—C8B—C9B	123.6 (3)	C21A—C22A—H22A	120.1
O2B—C8B—C9B	118.1 (3)	C23A—C22A—H22A	120.1
C1A—C9A—C8A	119.6 (3)	C23B—C22B—C21B	120.5 (3)
C1A—C9A—C10A	123.2 (3)	C23B—C22B—H22B	119.8
C8A—C9A—C10A	117.2 (2)	C21B—C22B—H22B	119.8
C1B—C9B—C8B	119.2 (3)	C22A—C23A—C24A	122.1 (3)
C1B—C9B—C10B	123.3 (3)	C22A—C23A—Cl1	118.7 (2)
C8B—C9B—C10B	117.4 (3)	C24A—C23A—Cl1	119.1 (2)
C11A—C10A—C25A	111.9 (2)	C22B—C23B—C24B	122.2 (3)
C11A—C10A—C9A	107.5 (2)	C22B—C23B—Cl2	119.4 (3)
C25A—C10A—C9A	114.5 (2)	C24B—C23B—Cl2	118.4 (3)
C11A—C10A—C26A	111.7 (2)	C25A—C24A—C23A	117.8 (3)
C25A—C10A—C26A	101.2 (2)	C25A—C24A—H24A	121.1
C9A—C10A—C26A	109.9 (2)	C23A—C24A—H24A	121.1
C9B—C10B—C11B	108.4 (2)	C25B—C24B—C23B	116.8 (3)
C9B—C10B—C25B	111.3 (2)	C25B—C24B—H24B	121.6
C11B—C10B—C25B	113.7 (2)	C23B—C24B—H24B	121.6
C9B—C10B—C26B	111.4 (2)	C24A—C25A—C20A	120.4 (3)
C11B—C10B—C26B	110.9 (2)	C24A—C25A—C10A	130.7 (3)
C25B—C10B—C26B	101.0 (2)	C20A—C25A—C10A	108.8 (2)
C19A—C11A—C12A	118.5 (3)	C24B—C25B—C20B	121.4 (3)
C19A—C11A—C10A	124.1 (3)	C24B—C25B—C10B	129.6 (3)
C12A—C11A—C10A	117.4 (3)	C20B—C25B—C10B	108.9 (3)
C19B—C11B—C12B	118.7 (3)	O6A—C26A—N1A	127.4 (3)
C19B—C11B—C10B	122.8 (3)	O6A—C26A—C10A	125.2 (3)
C12B—C11B—C10B	118.5 (3)	N1A—C26A—C10A	107.5 (2)
O5A—C12A—O3A	117.9 (3)	O6B—C26B—N1B	127.6 (3)
O5A—C12A—C11A	124.1 (3)	O6B—C26B—C10B	124.3 (3)
O3A—C12A—C11A	118.0 (3)	N1B—C26B—C10B	108.0 (2)
O5B—C12B—O3B	116.7 (3)	C26A—N1A—C20A	112.9 (2)
O5B—C12B—C11B	124.6 (3)	C26A—N1A—H1A	123.6
O3B—C12B—C11B	118.7 (3)	C20A—N1A—H1A	123.6
O3A—C13A—C14A	117.2 (3)	C26B—N1B—C20B	112.2 (3)
O3A—C13A—C18A	121.3 (3)	C26B—N1B—H1B	123.9
C14A—C13A—C18A	121.5 (3)	C20B—N1B—H1B	123.9
O3B—C13B—C18B	121.5 (3)	C1A—O1A—C19A	117.0 (2)
O3B—C13B—C14B	116.9 (3)	C1B—O1B—C19B	117.3 (2)
C18B—C13B—C14B	121.6 (3)	C7A—O2A—C8A	122.3 (2)
C15A—C14A—C13A	118.8 (3)	C8B—O2B—C7B	121.6 (2)
C15A—C14A—H14A	120.6	C13A—O3A—C12A	121.9 (2)
C13A—C14A—H14A	120.6	C12B—O3B—C13B	121.8 (2)
			
C9A—C1A—C2A—C7A	−1.0 (4)	C16B—C17B—C18B—C19B	−179.6 (3)
O1A—C1A—C2A—C7A	178.5 (2)	C12A—C11A—C19A—O1A	−176.2 (3)
C9A—C1A—C2A—C3A	−179.9 (3)	C10A—C11A—C19A—O1A	5.3 (5)
O1A—C1A—C2A—C3A	−0.4 (4)	C12A—C11A—C19A—C18A	4.1 (4)
C9B—C1B—C2B—C7B	0.5 (4)	C10A—C11A—C19A—C18A	−174.4 (3)
O1B—C1B—C2B—C7B	−178.2 (2)	C13A—C18A—C19A—C11A	2.7 (4)
C9B—C1B—C2B—C3B	177.4 (3)	C17A—C18A—C19A—C11A	−177.9 (3)
O1B—C1B—C2B—C3B	−1.3 (4)	C13A—C18A—C19A—O1A	−177.1 (3)
C7A—C2A—C3A—C4A	−0.7 (4)	C17A—C18A—C19A—O1A	2.4 (4)
C1A—C2A—C3A—C4A	178.2 (3)	C12B—C11B—C19B—O1B	176.9 (2)
C7B—C2B—C3B—C4B	0.4 (5)	C10B—C11B—C19B—O1B	−3.6 (4)
C1B—C2B—C3B—C4B	−176.4 (3)	C12B—C11B—C19B—C18B	−2.4 (4)
C2A—C3A—C4A—C5A	0.4 (5)	C10B—C11B—C19B—C18B	177.2 (3)
C2B—C3B—C4B—C5B	−0.3 (5)	C13B—C18B—C19B—C11B	2.7 (4)
C3A—C4A—C5A—C6A	0.2 (5)	C17B—C18B—C19B—C11B	−177.0 (3)
C3B—C4B—C5B—C6B	0.0 (5)	C13B—C18B—C19B—O1B	−176.6 (2)
C4A—C5A—C6A—C7A	−0.5 (5)	C17B—C18B—C19B—O1B	3.6 (4)
C4B—C5B—C6B—C7B	0.1 (5)	N1A—C20A—C21A—C22A	−178.6 (3)
C5A—C6A—C7A—O2A	−179.6 (3)	C25A—C20A—C21A—C22A	0.2 (4)
C5A—C6A—C7A—C2A	0.2 (5)	C25B—C20B—C21B—C22B	2.4 (5)
C3A—C2A—C7A—O2A	−179.8 (3)	N1B—C20B—C21B—C22B	−176.6 (3)
C1A—C2A—C7A—O2A	1.2 (4)	C20A—C21A—C22A—C23A	−0.2 (4)
C3A—C2A—C7A—C6A	0.4 (4)	C20B—C21B—C22B—C23B	0.5 (5)
C1A—C2A—C7A—C6A	−178.6 (3)	C21A—C22A—C23A—C24A	−0.6 (5)
C5B—C6B—C7B—O2B	179.6 (3)	C21A—C22A—C23A—Cl1	179.0 (2)
C5B—C6B—C7B—C2B	0.1 (5)	C21B—C22B—C23B—C24B	−2.9 (5)
C3B—C2B—C7B—O2B	−179.8 (3)	C21B—C22B—C23B—Cl2	176.5 (3)
C1B—C2B—C7B—O2B	−2.7 (4)	C22A—C23A—C24A—C25A	1.4 (4)
C3B—C2B—C7B—C6B	−0.4 (4)	Cl1—C23A—C24A—C25A	−178.2 (2)
C1B—C2B—C7B—C6B	176.7 (3)	C22B—C23B—C24B—C25B	2.2 (5)
O1A—C1A—C9A—C8A	−179.9 (3)	Cl2—C23B—C24B—C25B	−177.2 (2)
C2A—C1A—C9A—C8A	−0.5 (4)	C23A—C24A—C25A—C20A	−1.4 (4)
O1A—C1A—C9A—C10A	−1.2 (5)	C23A—C24A—C25A—C10A	175.3 (3)
C2A—C1A—C9A—C10A	178.2 (3)	C21A—C20A—C25A—C24A	0.6 (4)
O4A—C8A—C9A—C1A	−178.5 (3)	N1A—C20A—C25A—C24A	179.6 (3)
O2A—C8A—C9A—C1A	1.7 (4)	C21A—C20A—C25A—C10A	−176.7 (3)
O4A—C8A—C9A—C10A	2.7 (4)	N1A—C20A—C25A—C10A	2.3 (3)
O2A—C8A—C9A—C10A	−177.0 (2)	C11A—C10A—C25A—C24A	−59.6 (4)
O1B—C1B—C9B—C8B	−177.7 (2)	C9A—C10A—C25A—C24A	63.1 (4)
C2B—C1B—C9B—C8B	3.8 (4)	C26A—C10A—C25A—C24A	−178.7 (3)
O1B—C1B—C9B—C10B	5.8 (4)	C11A—C10A—C25A—C20A	117.4 (3)
C2B—C1B—C9B—C10B	−172.8 (3)	C9A—C10A—C25A—C20A	−119.9 (3)
O4B—C8B—C9B—C1B	175.1 (3)	C26A—C10A—C25A—C20A	−1.7 (3)
O2B—C8B—C9B—C1B	−5.9 (4)	C23B—C24B—C25B—C20B	0.8 (4)
O4B—C8B—C9B—C10B	−8.2 (4)	C23B—C24B—C25B—C10B	177.1 (3)
O2B—C8B—C9B—C10B	170.8 (2)	C21B—C20B—C25B—C24B	−3.1 (5)
C1A—C9A—C10A—C11A	5.6 (4)	N1B—C20B—C25B—C24B	176.0 (3)
C8A—C9A—C10A—C11A	−175.7 (2)	C21B—C20B—C25B—C10B	179.9 (3)
C1A—C9A—C10A—C25A	−119.4 (3)	N1B—C20B—C25B—C10B	−1.0 (3)
C8A—C9A—C10A—C25A	59.3 (3)	C9B—C10B—C25B—C24B	−56.8 (4)
C1A—C9A—C10A—C26A	127.4 (3)	C11B—C10B—C25B—C24B	65.9 (4)
C8A—C9A—C10A—C26A	−53.9 (3)	C26B—C10B—C25B—C24B	−175.2 (3)
C1B—C9B—C10B—C11B	−9.3 (4)	C9B—C10B—C25B—C20B	119.8 (3)
C8B—C9B—C10B—C11B	174.1 (2)	C11B—C10B—C25B—C20B	−117.4 (3)
C1B—C9B—C10B—C25B	116.4 (3)	C26B—C10B—C25B—C20B	1.5 (3)
C8B—C9B—C10B—C25B	−60.2 (3)	C11A—C10A—C26A—O6A	61.6 (4)
C1B—C9B—C10B—C26B	−131.7 (3)	C25A—C10A—C26A—O6A	−179.2 (3)
C8B—C9B—C10B—C26B	51.8 (3)	C9A—C10A—C26A—O6A	−57.7 (4)
C25A—C10A—C11A—C19A	119.0 (3)	C11A—C10A—C26A—N1A	−118.6 (3)
C9A—C10A—C11A—C19A	−7.6 (4)	C25A—C10A—C26A—N1A	0.6 (3)
C26A—C10A—C11A—C19A	−128.3 (3)	C9A—C10A—C26A—N1A	122.1 (3)
C25A—C10A—C11A—C12A	−59.5 (3)	C9B—C10B—C26B—O6B	60.6 (4)
C9A—C10A—C11A—C12A	173.9 (2)	C11B—C10B—C26B—O6B	−60.3 (4)
C26A—C10A—C11A—C12A	53.2 (3)	C25B—C10B—C26B—O6B	178.8 (3)
C9B—C10B—C11B—C19B	8.3 (4)	C9B—C10B—C26B—N1B	−119.8 (3)
C25B—C10B—C11B—C19B	−116.1 (3)	C11B—C10B—C26B—N1B	119.3 (3)
C26B—C10B—C11B—C19B	130.9 (3)	C25B—C10B—C26B—N1B	−1.6 (3)
C9B—C10B—C11B—C12B	−172.2 (2)	O6A—C26A—N1A—C20A	−179.5 (3)
C25B—C10B—C11B—C12B	63.5 (3)	C10A—C26A—N1A—C20A	0.7 (3)
C26B—C10B—C11B—C12B	−49.6 (3)	C21A—C20A—N1A—C26A	176.9 (3)
C19A—C11A—C12A—O5A	170.8 (3)	C25A—C20A—N1A—C26A	−1.9 (4)
C10A—C11A—C12A—O5A	−10.6 (4)	O6B—C26B—N1B—C20B	−179.3 (3)
C19A—C11A—C12A—O3A	−9.7 (4)	C10B—C26B—N1B—C20B	1.1 (3)
C10A—C11A—C12A—O3A	168.8 (3)	C21B—C20B—N1B—C26B	179.0 (3)
C19B—C11B—C12B—O5B	−178.1 (3)	C25B—C20B—N1B—C26B	−0.1 (4)
C10B—C11B—C12B—O5B	2.3 (4)	C9A—C1A—O1A—C19A	−2.2 (4)
C19B—C11B—C12B—O3B	0.6 (4)	C2A—C1A—O1A—C19A	178.3 (2)
C10B—C11B—C12B—O3B	−179.0 (2)	C11A—C19A—O1A—C1A	0.2 (4)
O3A—C13A—C14A—C15A	−177.2 (3)	C18A—C19A—O1A—C1A	179.9 (2)
C18A—C13A—C14A—C15A	2.1 (5)	C9B—C1B—O1B—C19B	0.4 (4)
O3B—C13B—C14B—C15B	−179.2 (3)	C2B—C1B—O1B—C19B	179.0 (2)
C18B—C13B—C14B—C15B	0.7 (5)	C11B—C19B—O1B—C1B	−1.4 (4)
C13A—C14A—C15A—C16A	0.1 (5)	C18B—C19B—O1B—C1B	177.9 (2)
C13B—C14B—C15B—C16B	0.6 (5)	C6A—C7A—O2A—C8A	179.8 (3)
C14A—C15A—C16A—C17A	−1.8 (5)	C2A—C7A—O2A—C8A	0.0 (4)
C14B—C15B—C16B—C17B	−1.2 (5)	O4A—C8A—O2A—C7A	178.7 (3)
C15A—C16A—C17A—C18A	1.2 (5)	C9A—C8A—O2A—C7A	−1.5 (4)
C15B—C16B—C17B—C18B	0.5 (5)	O4B—C8B—O2B—C7B	−177.1 (3)
O3A—C13A—C18A—C17A	176.6 (3)	C9B—C8B—O2B—C7B	3.8 (4)
C14A—C13A—C18A—C17A	−2.7 (5)	C6B—C7B—O2B—C8B	−178.9 (3)
O3A—C13A—C18A—C19A	−4.0 (4)	C2B—C7B—O2B—C8B	0.5 (4)
C14A—C13A—C18A—C19A	176.8 (3)	C14A—C13A—O3A—C12A	177.4 (3)
C16A—C17A—C18A—C13A	1.0 (4)	C18A—C13A—O3A—C12A	−1.9 (4)
C16A—C17A—C18A—C19A	−178.4 (3)	O5A—C12A—O3A—C13A	−171.7 (3)
O3B—C13B—C18B—C17B	178.6 (3)	C11A—C12A—O3A—C13A	8.8 (4)
C14B—C13B—C18B—C17B	−1.4 (4)	O5B—C12B—O3B—C13B	179.6 (3)
O3B—C13B—C18B—C19B	−1.2 (4)	C11B—C12B—O3B—C13B	0.8 (4)
C14B—C13B—C18B—C19B	178.9 (3)	C18B—C13B—O3B—C12B	−0.5 (4)
C16B—C17B—C18B—C13B	0.7 (4)	C14B—C13B—O3B—C12B	179.4 (3)

### Hydrogen-bond geometry (Å, º)

*Cg*1, *Cg*2 and *Cg*3 are the centroids of the C13*B*–C18*B*, C2*B*–C7*B* and C20*B*–C25*B* rings, respectively.

**Table:**

*D*—H···*A*	*D*—H	H···*A*	*D*···*A*	*D*—H···*A*
C22*B*—H22*B*···O4*A*	0.93	2.41	3.218 (4)	146
N1*A*—H1*A*···O6*B*^i^	0.86	2.08	2.810 (3)	142
N1*B*—H1*B*···O5*B*^i^	0.86	2.12	2.968 (3)	169
C5*A*—H5*A*···O4*B*^ii^	0.93	2.58	3.227 (4)	127
C5*B*—H5*B*···O5*A*^iii^	0.93	2.27	3.196 (4)	173
C14*B*—H14*B*···O4*B*^iv^	0.93	2.55	3.035 (4)	113
C17*A*—H17*A*···O6*A*^v^	0.93	2.49	3.312 (4)	148
C24*A*—H24*A*···Cl2^vi^	0.93	2.69	3.520 (3)	149
C4a—H4a···*Cg*1^vi^	0.93	2.95	3.832 (4)	158
C6a—H6a···*Cg*2^vi^	0.93	2.74	3.657 (4)	169
C5a—H5a···*Cg*3^ii^	0.93	2.88	3.701 (3)	147

Symmetry codes: (i) −*x*+1, −*y*+1, −*z*+1; (ii) −*x*, *y*−1/2, −*z*+1/2; (iii) *x*, −*y*+3/2, *z*−1/2; (iv) *x*, −*y*+3/2, *z*+1/2; (v) −*x*, −*y*, −*z*+1; (vi) −*x*, −*y*+1, −*z*+1.
